# Supplementary material for: Dietary Interventions of Salmon and Silver Carp Phospholipids on Mice with Metabolic Syndrome Based on Lipidomics
Source: Cells. 2022 Oct 12;11(20):3199. doi: 10.3390/cells11203199 (PMC9601277; doi:10.3390/cells11203199)
Supplement: Supplementary file 1 [file cells-11-03199-s001.zip › Supplementary Table S1. The feed ingredients.pdf]

Supplementary Table S1

Ingredient (g kg $^{-1}$)	LF	HF	SA4	SA2	HM4	HM2
Casein	200	200	200	200	200	200
Corn starch	600	250	210	230	210	230
Fructose	-	200	200	200	200	200
Corn oil	50	50	50	50	50	50
Lard	-	150	150	150	150	150
Sesame oil	50	50	50	50	50	50
Mineral	35	35	35	35	35	35
Vitamin	10	10	10	10	10	10
Cellulose	50	50	50	50	50	50
Choline bitartrate	3	3	3	3	3	3
DL- Methionine	2	2	2	2	2	2
Salmon phospholipid	-	-	40	20	-	-
silver carp phospholipid	-	-	-	-	40	20
Total	1000	1000	1000	1000	1000	1000
Energy (Kcal g $^{-1}$)	3.86	4.86	4.86	4.86	4.86	4.86

Note: Feed ingredients in animal experiment
