# Supplementary figures and images for: Dietary Interventions of Salmon and Silver Carp Phospholipids on Mice with Metabolic Syndrome Based on Lipidomics
Source: Cells. 2022 Oct 12;11(20):3199. doi: 10.3390/cells11203199 (PMC9601277; doi:10.3390/cells11203199)

Supplementary Figure S1: histological examination of the mice liver tissue.

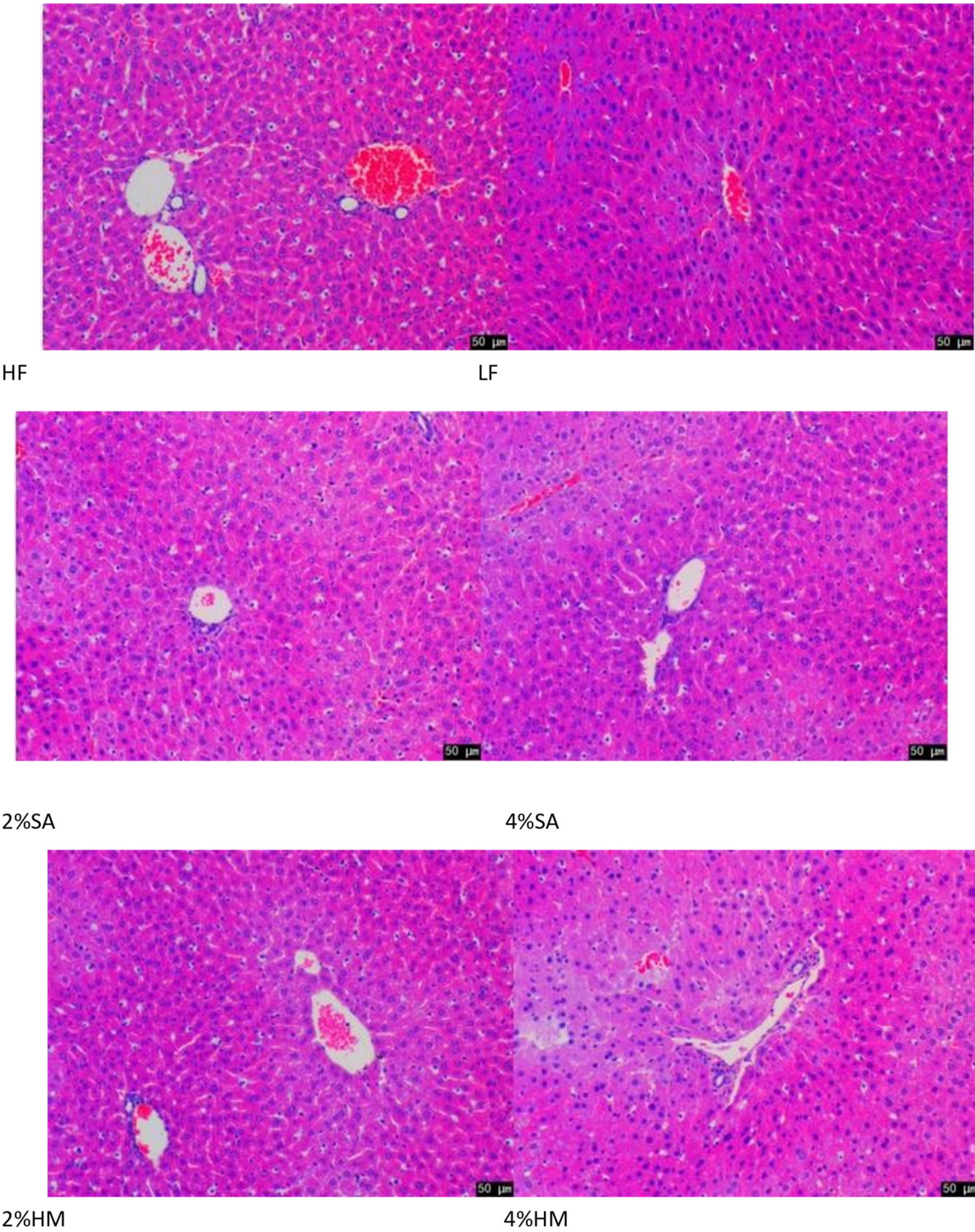

Supplement: Supplementary file 1 [file cells-11-03199-s001.zip › Supplementary Figure S1. Histological examination of the mice liver tissue.pdf]
